# Supplementary figures and images for: Identification of Plasmodium falciparum circumsporozoite protein-specific CD8+ T cell epitopes in a malaria exposed population
Source: PLoS One. 2020 Feb 10;15(2):e0228177. doi: 10.1371/journal.pone.0228177 (PMC7010280; doi:10.1371/journal.pone.0228177)

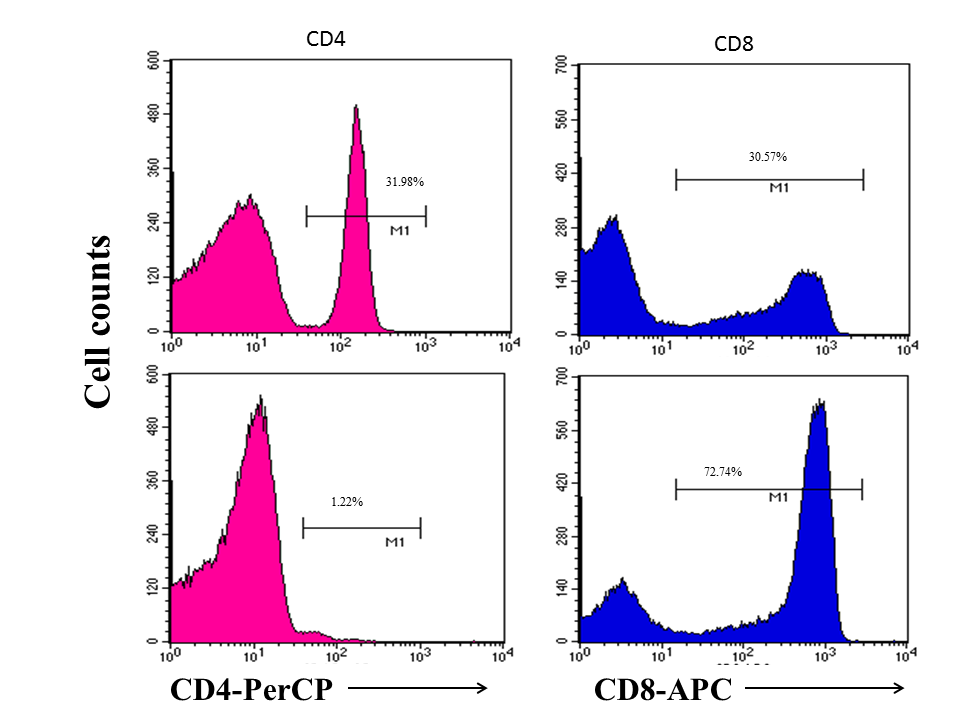

Supplement: S1 Fig — PBMCs from eight of the nine study subjects were depleted of cells expressing the CD4 receptor for the purpose of assessing the T cell lineage of peptide-specific IFN- responses. PBMC from all such depletions showed very similar results. Proportions indicate the percentage of total cells gated. (TIF) [file pone.0228177.s001.tif]
