# Supplementary material for: Identification of Plasmodium falciparum circumsporozoite protein-specific CD8+ T cell epitopes in a malaria exposed population
Source: PLoS One. 2020 Feb 10;15(2):e0228177. doi: 10.1371/journal.pone.0228177 (PMC7010280; doi:10.1371/journal.pone.0228177)
Supplement: Attachment — Submitted filename: Response to reviewers.docx [file pone.0228177.s002.docx]

**Responses to reviewer comments**

We thank the reviewer for the very insightful comments, and have provided a point-by-point response to these below;

Reviewer #1: This manuscript addresses the fine mapping of CD8 T cell epitopes on the P. falciparum Circumsporozoite Protein recognised by PBMCs from naturally-exposed Ghanaian donors. This forms a continuation of previous work by the authors in which they already demonstrated recognition of larger peptide pools spanning these epitopes by Ghanaian donors; the majority of these epitopes are also recognised by sporozoite vaccinees. Experimental validation of epitope prediction algorithms is crucial prior to such epitopes being selected for use in a multi-epitope sub-unit pre-erythrocytic malaria vaccine, which may form an alternative to attenuated whole-sporozoite approaches.

*As proof of principle, the authors identify several such CD8-restricted epitopes, which moreover appear to be conserved across P.f. strains and may be recognised by multiple HLA supertypes (both of which are advantageous for inclusion in a vaccine). Unfortunately, fewer predicted peptides than perhaps expected could be conclusively shown to be recognised (particularly in the 'CD8-enriched' cultures, see also below). It is also perhaps slightly concerning that the 'HLA-promiscuous' epitopes are not recognised more broadly. Finally, as the authors acknowledge, association of any of these epitopes with protection remains to be demonstrated.*

**Response:** Only a very limited number of peptides (between 3 and 8) were tested against PBMCs from each of the 9 study volunteers (due to limited cell numbers). We therefore did not expect to identify many epitopes per individual. What we sought to show is how many of the positive peptides with whole PBMCs are HLA class I-restricted, since there is also the possibility of having some of the immunodominant epitopes to be HLA class II-restricted.

The HLA diversity in these 9 volunteers may be too limited to actually demonstrate binding promiscuity. It is also important to note that promiscuity in HLA binding as described in the manuscript is mostly based on predictive algorithms which have been duly referenced and not necessarily on experimental data.

Regarding the statement on association with protection, we used PBMCs from adult volunteers with a history of natural exposure to malaria infections, but we did not have any information on their antimalarial protection status, hence that statement. We are currently working on addressing this, in a study where we have recruited people with a history of recent exposure to infectious bites and sometimes even show malaria parasites in the blood (an indication of a completed liver stage cycle) but with no clinical symptoms of malaria. Thus in our current on-going study, we have defined “protection” as evidence of exposure to recent infectious bites that do not result in clinical malaria symptoms.

The study approach is generally clear and appropriate. The manuscript is well-written and the abstract and discussion balanced, including addressing unexpected results and most limitations. Ethical approval is in place.

*The immunological methodology does suffer from some limitations, which presumably can no longer be addressed experimentally, but could perhaps be addressed a little more extensively in the discussion.*

**Response**: We have included additional study limitations and how they impact the presented data to the discussion section.

*Although highly suggestive, the depletion of CD4+ T cells does not conclusively prove that the remaining IFNg must be due to CD8+ T cells. Did the authors not consider depleting CD8-expressing cells in first instance instead of CD4-expressing cells, in order to directly demonstrate CD8+ T cells' role? An obvious alternative route would have been flow cytometry, but presumably this was not available?*

**Response**: We initially considered testing both CD8-enriched and CD4 enriched T cells for comparison with whole PBMCs, but had to go with just one of them because of the limited cell numbers

*What kit exactly was used for negative selection of CD8+ T cells? The M&M section mentions variously '[depleting] all cell types expressing the CD4 receptor' and 'a cocktail of ... antibodies against non-CD8+ T cells'. Depending on this, DCs, monocytes (both of which may also express CD4) and/or B-cells may have been depleted from the PBMCs in addition to CD4+ T cells, limiting the availability of APCs to (cross-)present the peptide to the remaining CD8+ T cells. May this partly explain the lower than expected sfc count in the 'CD8-enriched' samples compared to whole PBMC samples for many of the peptides (which were all supposed to be MHC-I restricted)? Were responses to CEF also lower in the 'CD8-enriched' samples?*

**Response**: We used the anti-human MyOne™ SA Dynabeads® kit (Invitrogen, Life Technologies) for CD8 T cell enrichment, and we agree with the reviewer regarding depletion of some antigen presenting cells (APCs) along with CD4 T cells since some APC subsets may also express the targeted receptors. It is however important to note that some other APC subsets likely to remain to undertake antigen cross-presentation, although we agree with the reviewer that these might have lowered the observed epitope positivity rate. This would then mean that our responses could have been stronger than observed, in the presence of the full complement of APCs.

All volunteers responded positively to at least one of the three positive controls

*The authors apply response positivity criteria used and validated in their previous studies. Nevertheless, given (perhaps not unexpectedly) the generally marginal responses to individual peptides (corrected 0-38 sfc/m) in comparison to the range of the neg control (1-24 sfc/m), could the authors in table 2 and 3 maybe provide for each subject the actual sfc value of the neg control for respectively whole PBMC and CD8-enriched cultures? Responses to individual peptides could then be shown either as absolute counts or, as currently, corrected counts. Either way, the reader will be able to form a slightly better impression of the relative strength of individual peptide responses. Were neg control responses generally also lower in the 'CD8-enriched' samples than the unfractionated samples? May this explain why 'positivity criteria' for e.g. SVFNVVNSSI were not met in the unfractionated PBMCs?*

 Response: We agree with the reviewer and have provided the respective medium/background control values in Tables 2 and 3. We have therefore reverted to absolute counts (without background subtraction) for each stimulant in these two tables. We have also incorporated data on the positive control and background counts for assays with CD8+ T cell enriched PBMCs in the results section.
